# Effects of Donepezil Treatment on Brain Metabolites, Gut Microbiota, and Gut Metabolites in an Amyloid Beta-Induced Cognitive Impairment Mouse Pilot Model

**DOI:** 10.3390/molecules27196591

**Published:** 2022-10-05

**Authors:** Jae-Kwon Jo, Gihyun Lee, Cong Duc Nguyen, Seong-Eun Park, Eun-Ju Kim, Hyun-Woo Kim, Seung-Ho Seo, Kwang-Moon Cho, Sun Jae Kwon, Jae-Hong Kim, Hong-Seok Son

**Affiliations:** 1 Department of Biotechnology, College of Life Sciences and Biotechnology, Korea University, Seoul 02841, Korea; 2 Department of Korean Medicine, Dongshin University, Naju 58245, Korea; 3 Sonlab Inc., Seoul 02841, Korea; 4 AccuGene Inc., Incheon 21999, Korea; 5 Department of Acupuncture and Moxibustion Medicine, College of Korean Medicine, Dongshin University, Naju 58245, Korea

**Keywords:** Alzheimer’s disease, gut microbiome, amyloid beta, donepezil, metabolite

## Abstract

Accumulated clinical and biomedical evidence indicates that the gut microbiota and their metabolites affect brain function and behavior in various central nervous system disorders. This study was performed to investigate the changes in brain metabolites and composition of the fecal microbial community following injection of amyloid β (Aβ) and donepezil treatment of Aβ-injected mice using metataxonomics and metabolomics. Aβ treatment caused cognitive dysfunction, while donepezil resulted in the successful recovery of memory impairment. The Aβ + donepezil group showed a significantly higher relative abundance of Verrucomicrobia than the Aβ group. The relative abundance of 12 taxa, including *Blautia* and *Akkermansia*, differed significantly between the groups. The Aβ + donepezil group had higher levels of oxalate, glycerol, xylose, and palmitoleate in feces and oxalate, pyroglutamic acid, hypoxanthine, and inosine in brain tissues than the Aβ group. The levels of pyroglutamic acid, glutamic acid, and phenylalanine showed similar changes in vivo and in vitro using HT-22 cells. The major metabolic pathways in the brain tissues and gut microbiota affected by Aβ or donepezil treatment of Aβ-injected mice were related to amino acid pathways and sugar metabolism, respectively. These findings suggest that alterations in the gut microbiota might influence the induction and amelioration of Aβ-induced cognitive dysfunction via the gut–brain axis. This study could provide basic data on the effects of Aβ and donepezil on gut microbiota and metabolites in an Aβ-induced cognitive impairment mouse model.

## 1. Introduction

Alzheimer’s disease (AD) is a neurodegenerative disorder characterized by progressive cognitive decline with impairment in several cognitive areas, including memory, executive function, and language. AD accounts for 60–80% of all dementia cases worldwide and is a leading cause of morbidity and mortality (alz.org/what-is-dementia.asp). Current major challenges in AD include the lack of reliable biomarkers for early diagnosis and effective prevention and treatment strategies [1]. Therefore, an increased understanding of the molecular etiology of AD may lead to the development of improved diagnostic and therapeutic strategies.

Metabolomics can be defined as the untargeted analysis of the composition of small-molecule metabolites that can capture changes across several physiological pathways driven by complex interactions between genetic and environmental risk factors [2]. Growing evidence suggests that AD is a pervasive metabolic disorder with dysregulation of multiple biochemical pathways in the brain, which may be associated with the severity of AD pathology [3]. Several metabolomic studies have examined the relationship between metabolism and AD pathology [4]. Although large-scale unbiased metabolomics techniques have been applied to understand AD pathology and its triggering symptoms, the scope of analysis has been limited to the brain itself. This is due to the conventional consideration that AD is bounded as a central nervous system (CNS) disorder. However, growing experimental, epidemiological, and clinical evidence suggests that the signs of AD extend beyond the brain [5].

The human gut consists of a very complex gut microbiome that is essential for maintaining the health of the host. Dysbiosis of the composition of the gut microbiome due to dietary changes and infection results in the loss of homeostasis, which is associated with the pathogenesis of several human diseases, including neurodegenerative disorders [6]. Accumulating clinical and biomedical evidence indicates that the gut microbial groups and their metabolites affect brain functions and behavior in various CNS disorders, including depression, cognitive decline, autism, and multiple sclerosis [7,8,9]. In particular, reduced microbial diversity has been reported to be associated with AD. Several studies have observed significant differences in the phylogenetic order, grade, and familial level between healthy controls and AD [10]. The human gut microbiota contributes to brain function via the cumulative effects of neuronal, humoral, and immune pathways as well as microbial metabolites. Remarkable genome-based metabolic modeling of the human gut microbiota has shown that several genera have the predictive capacity to produce or consume neurotransmitters, such as γ-aminobutyric acid (GABA) and serotonin, which play important roles in regulating brain function [11]. Although it has become increasingly clear that metabolite activity in the gut microbiota provides a mechanistic association with brain function and behavior, only a few studies have examined the complex relationship between brain metabolites and gut microbiota. Therefore, it is not yet known how gut microbes interact with brain metabolites in the process of promoting or preventing AD. Bacterial profiles of transgenic APP/PS1 mice, a well-established deterministic mouse model of AD, showed significant differences between wild-type and transgenic mice. In particular, the deposits of amyloid β (Aβ) in the brain coincided with the onset of showing differences in the gut microbiota [12,13,14]

Extracellular accumulation of Aβ plaques is a pathological hallmark of AD. Clinical and experimental evidence has suggested that a sharp increase in Aβ levels in the brain can lead to the development of an AD-like phenotype [15]. Animal models are being developed to replicate the symptoms, lesions, or causes of AD. To date, no complete animal model has emerged for AD, but various animal models have contributed significantly to our understanding of the molecular mechanisms of AD and memory deficits, which are intractable in human studies and impossible to check for in cultured cells [16]. Donepezil, an acetylcholinesterase inhibitor (AChEI), has been widely used to treat AD [17]. However, there are few studies on the effects of treatment with Aβ and donepezil, which are frequently used in Aβ-induced cognitive impairment animal models, on the gut microbiota and metabolites. Therefore, we aimed to investigate the changes in brain metabolites and fecal microbial community after treatment with Aβ and donepezil using metataxonomics and metabolomics.

## 2. Results

### 2.1. Effect of Donepezil on Aβ_25–35_ Memory Impairment

**Figure 1:**
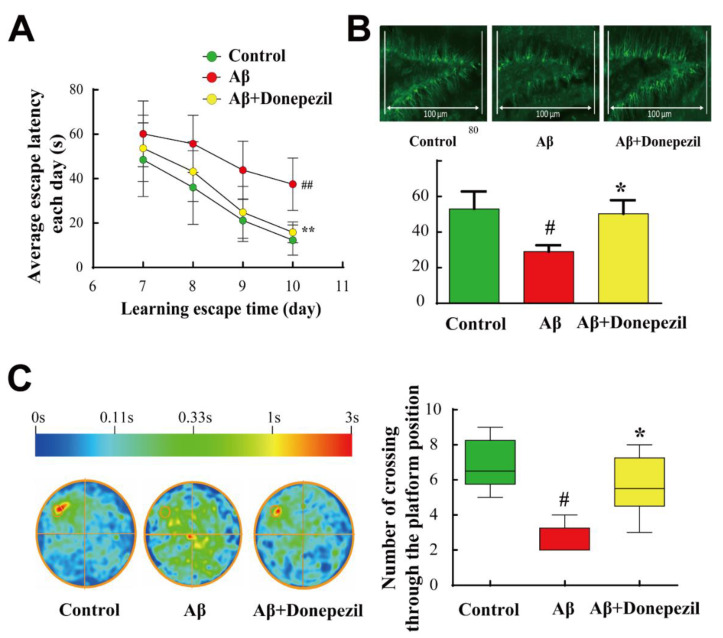
(**A**) Morris water maze test. The escape latency of mice on days 7–10 was recorded. (**B**) Doublecortin-positive cells in the dentate gyrus region. (**C**) Number of platform area crossings was determined by conducting a probe test at the end of day 10. The color scale indicates the average position distribution time of animals within each group. Data are presented as the mean ± standard error of the mean values of sextuple determinations. #, *p* < 0.05; ##, *p* < 0.01 vs. control group; *, *p* < 0.05; **, *p* < 0.01 vs. amyloid β (Aβ) group.

The Morris water maze (MWM) test was performed sequentially for four days. Donepezil treatment of Aβ-injected mice significantly reduced escape latency and the number of platform area crossings in mice compared to the Aβ group. Donepezil treatment of Aβ-injected mice resulted in a 3-fold decrease in escape latency of mice on the 10th day and a 2-fold increase in the crossing time in the probe test compared to Aβ_25–35_ treatment only, implying the successful elevation of memory performance by donepezil against Aβ_25–35_ induced neurodegeneration (Figure 1A). The spatial learning and memory ability of mice is closely related to the physiology of the hippocampus, which is essential for the neuroprocessing ability of mice [18]. Among the many anatomical parts of the hippocampus, the dentate gyrus is commonly studied and plays a key role in the formation, recall, and discrimination of episodic memory [19,20].

Therefore, we selected the dentate gyrus region to study the physiology of neuronal cells in the hippocampal region and determined the effect of donepezil on an anatomical scale. As doublecortin is a common marker of neurogenesis, this antibody was used to survey neuronal conditions in the dentate gyrus. The results showed that Aβ_25–35_ intracerebroventricular (ICV) injection decreased the number of doublecortin-positive cells in the dentate gyrus, whereas donepezil treatment of Aβ-injected mice increased it by 2-fold (Figure 1B). The results of crossover time in the probe test showed that Aβ_25–35_ treatment caused cognitive dysfunction, and donepezil resulted in the successful recovery of memory impairment (Figure 1C).

### 2.2. Composition of the Feces Microbiome

**Figure 2:**
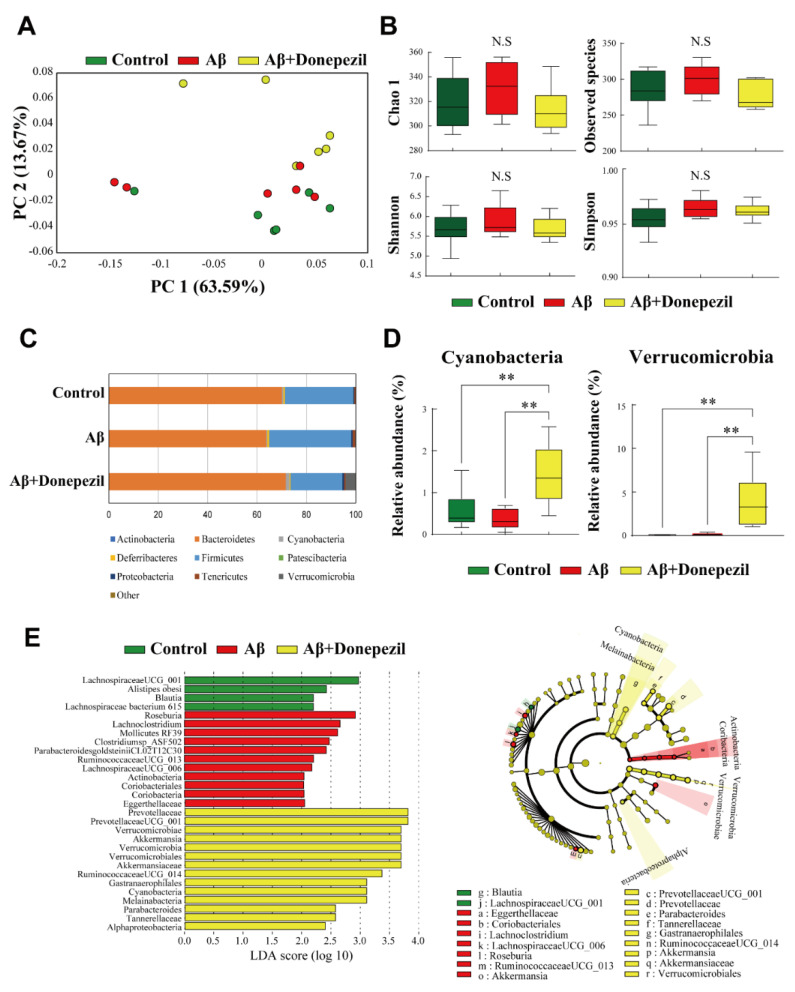
(**A**) Beta diversity analysis of the control, Aβ, and Aβ + donepezil groups based on weighted UniFrac distance matrices. (**B**) Alpha diversity analysis of the control, Aβ, and Aβ + donepezil groups. (**C**) Comparison of microbiota composition at the phylum level. (**D**) Relative abundance of cyanobacteria and Verrucomicrobia. (**E**) Linear discriminant analysis (LDA) and the cladogram show the phylogenetic distribution of microbes that are associated with the control, Aβ, and Aβ + donepezil groups. Taxonomic levels of the phylum, class, and order are labeled, while family and genus are abbreviated. Plots are represented using LDA effect size (LEfSe). **, *p* < 0.01.

Bacterial 16S rRNA gene sequencing of the fecal samples was performed to determine whether each treatment affected the gut microbiota. Beta diversity was assessed by principal coordinate analysis (PCoA) based on weighted UniFrac distance matrices. Aβ and donepezil treatment of Aβ-injected mice did not largely affect the overall composition of the gut microbiota (Figure 2A). The alpha diversity indices for the observed species, Shannon, Simpson, and Chao1 (richness and evenness), did not differ significantly between the groups (Figure 2B).

The relative abundance profile of bacterial communities at the phylum, genus, and species level is provided in Table S1. The Aβ + donepezil group showed a significantly higher relative abundance of cyanobacteria and Verrucomicrobia than the Aβ group (Figure 2C,D) (*p* < 0.01). The differentially abundant taxa were further confirmed by linear discriminant analysis effect size (LEfSe), which exploits linear discriminant analysis (LDA) to identify features that are statistically different among classes [21]. The resulting cladogram revealed that *Blautia* and *Lachnospiraceae* UCG_001 were more dominant in the control group than in the other groups, whereas *Eggerthellaceae*, *Coriobacteriales*, *Lachnoclostridium*, *Lachnospiraceae* UCG_006, *Roseburia*, *Ruminococcaeceae* UCG_013, and *Mollicutes* RF39 were more dominant in the Aβ group, and *Prevotellaceae* UCG_001, *Prevotellaceae*, *Parabacteroides*, *Tannerellaceae*, *Ruminococcaeceae* UCG_014, *Akkermansia*, *Akkermansiaceae*, *Gastranaerophilales*, and *Verrucomicrobiales* were more dominant in the Aβ + donepezil group (Figure 2E).

**Figure 3:**
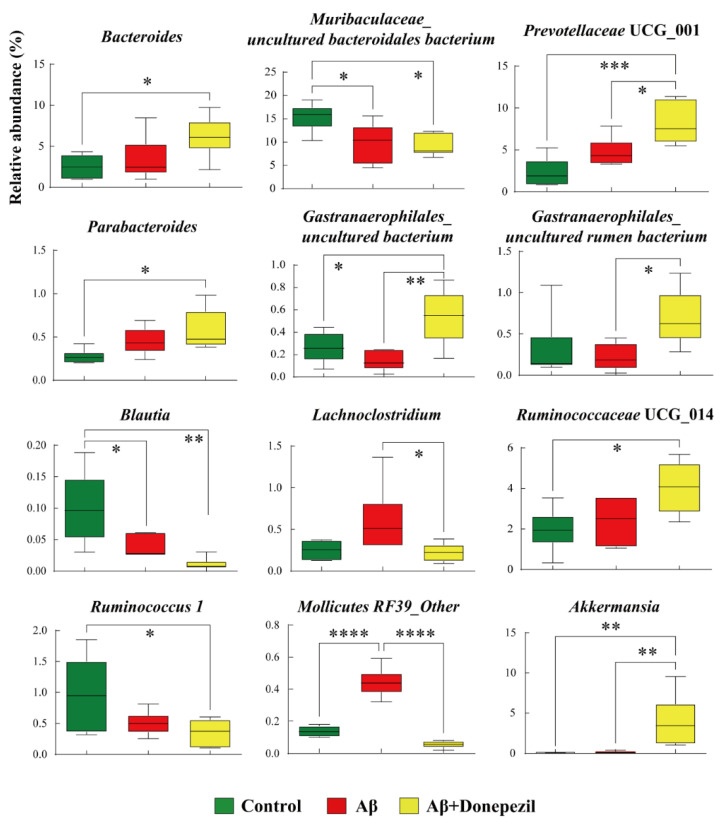
Box plots of significantly different microorganisms at the genus level in the feces of the control, Aβ, and Aβ + donepezil groups. The *p* values were obtained using one-way analysis of variance (ANOVA) with Tukey’s posthoc test for differences between groups; *, *p* < 0.05; **, *p* < 0.01; ***, *p* < 0.001; ****, *p* < 0.0001. A false discovery rate (FDR) of 5% was applied to all tests to correct for multiple testing.

Significant differences at the genus level in the gut bacteria of the control, Aβ, and Aβ + donepezil groups are shown in Figure 3. The relative abundances of 12 taxa (Bacteroides, *Muribaculaceae*_uncultured *Bacteroidales* bacterium, *Prevotellaceae* UCG_001, *Parabacteroides*, *Gastranaerophilales*_uncultured bacterium, *Gastranaerophilales*_uncultured rumen bacterium, *Blautia*, *Lachnoclostridium*, *Ruminococcaeceae* UCG_014, *Ruminococcus* 1, *Mollicutes* RF39, and *Akkermansia*) were significantly different between the groups. Interestingly, the relative abundance of *Blautia* was significantly higher in the control group than in the Aβ group, while the relative abundance of *Muribaculaceae*_uncultured *Bacteroidales* bacterium and *Mollicutes* RF39 were higher in the Aβ group than in the control group. This result suggests that the accumulation of Aβ_25–35_ in the brain might affect the gut microbiota. The relative abundances of *Prevotellaceae* UCG_001, *Gastranaerophilales*_uncultured bacterium, *Gastranaerophilales*_uncultured rumen bacterium, *Ruminococcaceae* UCG_014, and *Akkermansia* did not show a significant difference between the control group and the Aβ group, but they were significantly higher in the Aβ + donepezil group than in the Aβ group. Conversely, the relative abundances of *Lachnoclostridium* and *Mollicutes* RF39 were higher in the Aβ group than in the Aβ + donepezil group.

### 2.3. Profiling of Feces Metabolites

**Figure 4:**
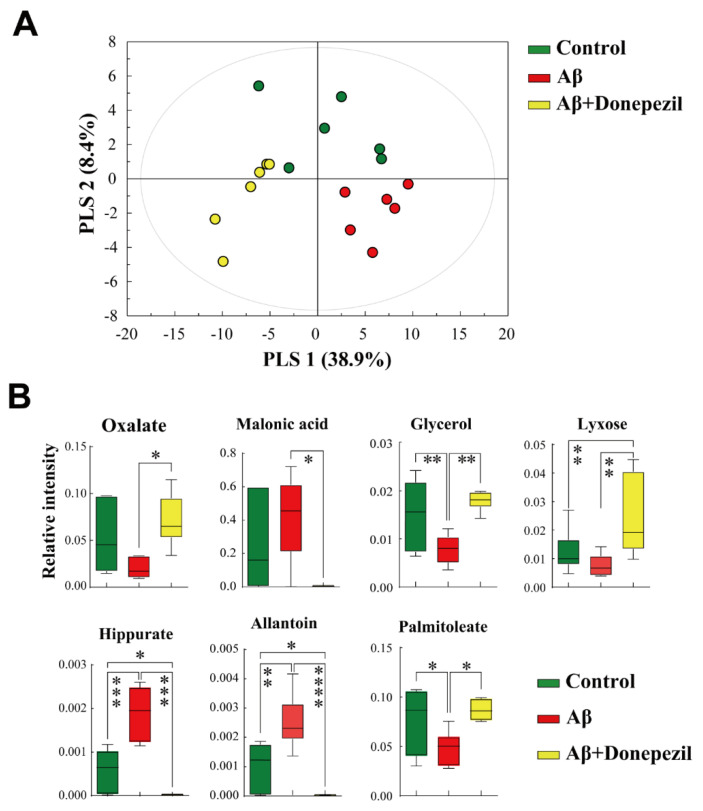
(**A**) Supervised partial least squares discriminant analysis (PLS-DA) score plot derived from gas chromatography-mass spectrometry (GC-MS) data of feces. (**B**) Box plots of significantly different metabolites in feces. *, *p* < 0.05; **, *p* < 0.01; ***, *p* < 0.001; ****, *p* < 0.0001. A false discovery rate (FDR) of 5% was applied to all tests to correct for multiple testing.

PLS-DA for supervised pattern recognition was applied to investigate different metabolite profiles and identify potential biomarkers (Figure 4A). The PLS-DA model showed a clear separation between the groups, suggesting that the changes in metabolites in the feces after each treatment differed between the groups. A permutation test supported the validity of the PLS-DA model. Among the 36 metabolites detected in fecal samples, those that significantly contributed to the discrimination were identified according to a threshold of VIP > 1.0, *p* < 0.05. The level of glycerol was significantly higher in the control group than that in the Aβ group (Figure 4B). The Aβ + donepezil group showed higher levels of oxalate, glycerol, xylose, and palmitoleate than the Aβ group, whereas the levels of malonic acid, hippurate, and allantoin were higher in the Aβ group than in the Aβ + donepezil group.

### 2.4. Profiling of Brain Tissue Metabolites

**Figure 5:**
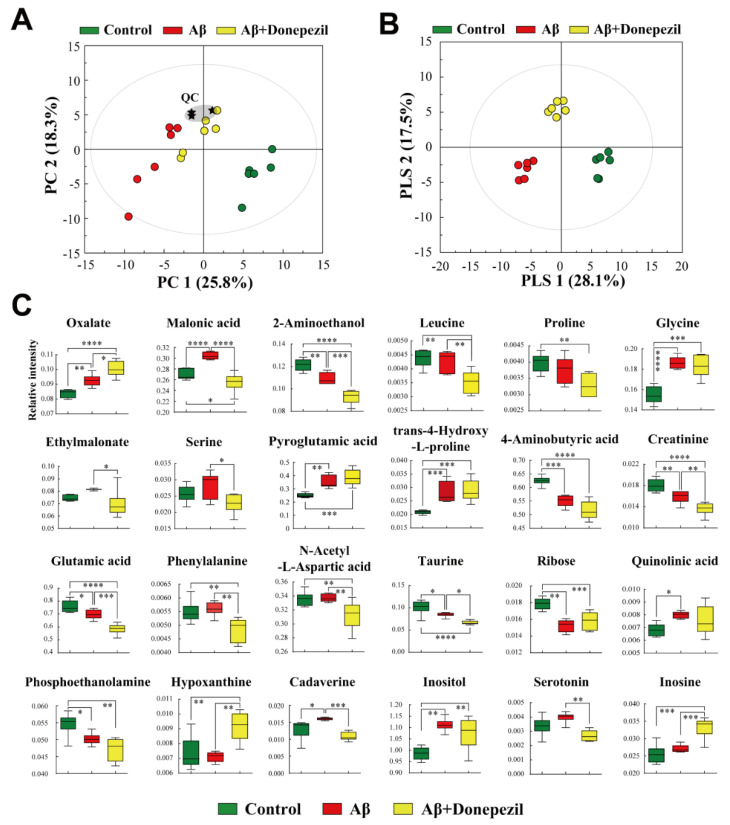
(**A**) Principal component analysis (PCA) score plot based on GC-MS data sets from brain tissues. (**B**) Supervised PLS-DA score plot derived from the GC-MS data from brain tissues. (**C**) Box plots of significantly different metabolites in the brain tissues. *, *p* < 0.05; **, *p* < 0.01; ***, *p* < 0.001; ****, *p* < 0.0001. A false discovery rate (FDR) of 5% was applied to all tests to correct for multiple testing.

PCA was performed to investigate the different metabolite profiles in the brain tissue (Figure 5A). In the PCA score plot, the Aβ and Aβ + donepezil groups were well separated from the control, indicating that there was a significant metabolite difference between the groups. To maximize the separation and identify potential biomarkers, PLS-DA was applied (Figure 5B). The PLS-DA score plot showed that the three groups were clearly separated from each other.

Variables that significantly contributed to the discrimination between the control and Aβ groups were selected based on a VIP > 1.0 and *p* < 0.05. Brain tissues injected with Aβ were characterized by higher levels of oxalate, malonic acid, glycine, pyroglutamic acid, trans-4-hydroxy-l-proline, quinolinic acid, cadaverine, and inositol (Figure 5C). Conversely, ethanolamine, GABA, creatinine, glutamic acid, taurine, and ribose levels were significantly higher in the control group than in the Aβ group. In the analysis between the Aβ group and the Aβ + donepezil group, the levels of oxalate, pyroglutamic acid, hypoxanthine, and inosine were significantly higher in the brain tissues of the Aβ + donepezil group than in the Aβ group. In contrast, the Aβ group was characterized by higher levels of malonic acid, ethanolamine, leucine, ethylmalonate, serine, creatinine, glutamic acid, taurine, cadaverine, phenylalanine, and serotonin than the Aβ + donepezil group. The leucine levels were significantly lower in the Aβ + donepezil group than in the Aβ group. Glutamate levels in the Aβ + donepezil group were lower than those in the Aβ group.

### 2.5. Profiling of HT-22 Cell Metabolites

The PLS-DA score plot revealed a separation pattern between the control, Aβ, Aβ + low donepezil, and Aβ + high donepezil groups, indicating that the metabolic profiles of the HT-22 cell samples differed between the groups (Figure S1A). The metabolites that significantly contributed to the discrimination between groups were selected based on a VIP > 1.0 and *p* < 0.05. Six potential biomarkers were identified: malic acid, pyroglutamic acid, glutamic acid, phenylalanine, glutamine, and tyrosine (Figure S1B). The levels of glutamic acid and glutamine were lower in the Aβ + high donepezil group than in the Aβ group. The levels of pyroglutamic acid, glutamic acid, and phenylalanine showed similar changes in vivo and in vitro using HT-22 cells. These results suggest that in both in vivo and in vitro settings, donepezil exhibits therapeutic effects through similar metabolic pathways. Moreover, this was an interesting result considering that donepezil was administered orally to mice but directly to cell culture. Wissmann et al. [22] reported that elevated phenylalanine concentrations were detected in clinical conditions accompanied by chronic inflammation and inflammation, which are associated with AD.

### 2.6. Metabolic Pathway Analysis

Metabolic pathway analysis was performed to identify the brain tissue and fecal metabolic pathways affected by each treatment (Figure S2). The metabolic pathways in brain tissue that were significantly affected by donepezil treatment of Aβ-injected mice were mainly amino acid pathways, including fatty acid biosynthesis, taurine and hypotaurine metabolism, tryptophan metabolism, arginine biosynthesis, and histidine metabolism. The metabolic pathways significantly affected by donepezil treatment of Aβ-injected mice in the fecal metabolic pathways are those related to sugar metabolism and include: glycerolipid metabolism, fatty acid biosynthesis, galactose metabolism, purine metabolism, and starch and sucrose metabolism.

### 2.7. Correlation between Microorganisms and Metabolites

**Figure 6:**
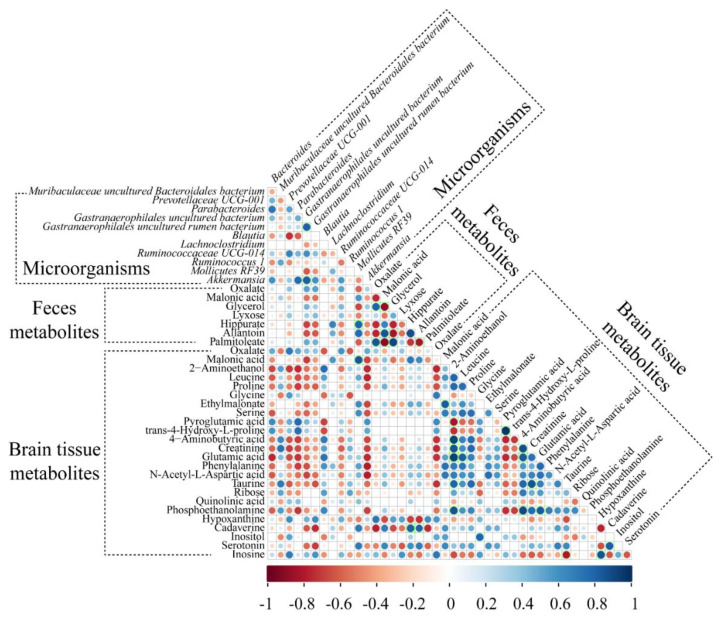
Heat map shows correlations (Spearman’s rank correlation, *p* < 0.05) among the identified brain tissue metabolites, feces metabolites, and feces microbiota. R-values of 0.8 or more are highlighted with green borders.

To understand the relationships between genus-level gut microorganisms and the metabolites of the brain tissue and feces, Spearman correlations were used to generate a heat map correlation matrix (|r| > 0.8) (Figure 6). The relative abundance of *Gastranaerophilales*_uncultured bacteria in feces was positively correlated with the relative abundances of *Gastranaerophilales*_uncultured rumen bacteria (r = 0.82) and *Akkermansia* (r = 0.82). The levels of 4-hydroxy-l-proline in the brain tissue correlated positively with the level of pyroglutamic acid in the brain tissue (r = 0.98). Inosine levels in brain tissue were positively correlated with hypoxanthine levels (r = 0.87). Palmitoleate levels in feces were positively correlated with oxalate (r = 0.86) and glycerol (r = 0.92) levels. Brain tissue taurine levels were positively correlated with the levels of creatinine in the brain tissue (r = 0.87). Brain tissue ethanolamine levels correlated positively with brain tissue creatinine levels and glutamic acid (r = 0.94) but negatively with the levels of pyroglutamic acid (r = −0.82) in brain tissue.

## 3. Discussion

The comprehensive etiology of AD remains unknown. Several hypotheses exist, such as neuritis, tau hyperphosphorylation, and Aβ plaques, but none fully explains the origin and progression of AD. In recent years, a growing body of research has suggested that there is a close relationship between gut microbiota and neurological disease called the gut-brain axis [23]. In the current study, microbial diversity (alpha and beta diversity) did not show a significant difference between the groups. Although high microbial diversity has traditionally been associated with better health conditions, some studies have reported controversial results. For example, recent studies reported that major active depressive disorder and autism are strongly related to microbiota composition and not to diversity [24,25]. However, ICV injection of Aβ_25–35_ significantly improved the relative abundance of Tenericutes in the Aβ group compared to that in the control group, suggesting that the bidirectional gut-brain axis affects the gut from the brain as well as the gut to the brain. Furthermore, Arnoriaga-Rodríguez et al. [26] revealed that an increase in the relative abundance of Tenericutes is positively associated with circulating Aβ, which is consistent with the results of the current study.

Although there was no significant difference between the control and Aβ groups, the relative abundance of Verrucomicrobia in the Aβ + donepezil group was significantly higher than that of the other groups. Additionally, at the genus level, the relative abundance of *Akkermansia*, a genus of Verrucomicrobia, was significantly higher in the Aβ + donepezil group than in the control and Aβ group. Recently, many studies reported that a reduction in the abundance of *Akkermansia* is associated with important risk factors for AD, including obesity and insulin resistance [27,28,29]. Ou et al. [30] reported that *Akkermansia muciniphila*, a representative strain of Verrucomicrobia, promoted the reduction of Aβ_40–42_ levels in the cerebral cortex of APP/PS1 mice. Furthermore, the author reported that *A. muciniphila* could prevent weight gain due to a high-fat diet, restore the impaired integrity of the intestinal epithelial barrier, reduce pro-inflammatory factors in the blood, and improve insulin resistance. In this study, the difference in serotonin levels was not significant between the control group and the Aβ group, but the Aβ + donepezil group showed a significantly lower level than the other groups. Several studies have reported that host-microbe interactions modulate the host serotonin or 5-hydroxytryptamine (5-HT) system [31,32]. Yaghoubfar et al. [33] reported the presence of *A. muciniphila* in the gut promotes serotonin concentration, which also affects serotonin signaling/metabolism through the gut-brain axis. Conversely, Shearman et al. [34] reported that donepezil treatment was effective in lowering serotonin levels. Therefore, the relationship between *Akkermansia* abundance and 5-HT levels is not clear. However, the increased abundance of *Akkermansia* in the Aβ + donepezil group suggests that oral administration of donepezil may have a positive effect on the abundance of *Akkermancia*. Cadaverine levels are altered in some neurological disorders, such as AD and Parkinson’s disease. Cadaverine is synthesized by the gut microbiome, suggesting that in the presence of this bacterial metabolite in the cerebrospinal fluid, it must cross the gut and brain barrier and be absorbed by the CNS. Vascellari et al. [35] reported that the increased level of cadaverine was positively correlated with the *Streptococcaceae* family and the related genus *Streptococcus*, which are known to express cadaverine biosynthetic enzymes. Another study suggested that cadaverine is involved in the inflammatory processes in AD [36]. Donepezil has recently been demonstrated to have anti-inflammatory effects against lipopolysaccharide and tau pathology [37]. In the current study, cadaverine in brain tissue showed a higher level in the Aβ group than in the control group and lower levels in the Aβ + donepezil group than in the Aβ group. Overall, the higher relative abundance of *Akkermansia* and the low cadaverine levels in the Aβ + donepezil group suggested that the anti-inflammatory effects of donepezil may be related to *Akkermansia* repair of the impaired integrity of the intestinal barrier. In this study, the relative abundances of *Prevotellaceae* UCG_001, *Ruminococcaceae* UCG_014, were not significantly different between the control group and the Aβ group but were significantly higher in the Aβ + donepezil group than in the Aβ group. Zhang et al. [38] reported that *Prevotellaceae* UCG_001 was found in AD treatment groups. Li et al. [39] reported that a decrease in *Prevotellaceae* is associated with memory deficits. *Ruminococcaceae* (*Ruminococcaceae* UCG_014 groups) were associated with behavioral changes induced by stress, and mice enriched in these taxa showed reduced epithelial oxidative and inflammatory damage [40,41]. Another study reported that *Ruminococcaceae* UCG_014 upregulates anti-inflammatory cytokines while downregulating pro-inflammatory molecules [42]. Therefore, their increase may indicate that it is related to the therapeutic effect for memory deficit.

In the current study, the relative abundance of *Blautia* was higher in the control group than in the Aβ group. Although animal studies regarding the effects of *Blautia* on AD have yielded conflicting results [43,44], one interesting finding was that the gut microbial neurotransmitter GABA, a product associated with *Blautia*-dependent arginine metabolism, was associated with a reduced risk of AD. In particular, GABA brain levels were reported to be decreased in the temporal cortex of AD patients in several case-control studies [45]. In the current study, the level of GABA was significantly lower in the Aβ group than in the control group (in vivo), but there was no difference in GABA in HT-22 cells (in vitro), suggesting that the correlation between GABA and *Blautia* might be a potential biomarker for Aβ-induced cognitive impairment.

In the analysis of brain tissue metabolites, brain tissues injected with Aβ were characterized by higher levels of quinolinic acid than that of the control group. Conversely, ethanolamine levels were significantly higher in the control group than in the Aβ group. Guillemin et al. [46] reported that Aβ_1–42_ induces the production of quinolinic acid at neurotoxic concentrations. Ethanolamine has various positive functions, ranging from cell signaling to molecular storage, and alterations in its levels have been linked to neurodegenerative conditions, such as AD. Gwanyanya et al. [47] reported that AD patients have 30–50% lower levels of ethanolamine in the brain. However, the levels of brain ethanolamine showed no significant difference between the Aβ + donepezil group and the Aβ group. In the analysis between the Aβ group and the Aβ + donepezil group, the level of hypoxanthine was significantly higher in brain tissues of the Aβ + donepezil group than the Aβ group. Hypoxanthine levels might reflect the cell cycle activity or cell death. In the current study, there was no significant difference between the control group and the Aβ group, but higher levels of hypoxanthine in the Aβ + donepezil group were consistent with other reports of higher plasma levels of hypoxanthine and a lower risk of incident AD [48]. Glutamate levels in the Aβ group were lower than in the control group. Furthermore, brain glutamate levels were lower in the Aβ + donepezil group than in the Aβ group. Although excitatory glutamatergic neurotransmission via N-methyl-d-aspartate (NMDA) receptors is critical for synaptic plasticity and neuronal survival, excessive NMDA receptor activity causes excitotoxicity and promotes cell death, underlying the potential mechanism of neurodegeneration in AD. Some previous studies have reported that decreased glutamate toxicity through the downregulation of NMDA receptors could be the mechanism underlying neuroprotection by donepezil [49,50]. Interestingly, in the current study, the malonic acid level was higher in the Aβ group than in the control group and lower in the Aβ + donepezil group compared to the Aβ group. Greene et al. [51] reported that the toxic effects of malonic acid induced neuronal death via NMDA receptor-mediated excitotoxicity. Therefore, the therapeutic effect of donepezil on Aβ-injected mice may be related to the reduction of glutamate and malonic acid levels associated with NMDA receptors.

In metabolism pathway analysis, Aβ aggregation and donepezil treatment of Aβ-injected mice had a significant effect on taurine and hypotaurine metabolism and glutamine and glutamate metabolism in brain tissue. Additionally, we observed that changes in the levels of glutamic acid and pyroglutamic acid had the opposite results. These patterns were observed in both HT-22 cells and the brain tissue. Li et al. [52] reported that soluble Aβ oligomers perturbed synaptic plasticity by altering glutamate recycling at the synapse and decreasing glutamate uptake. Furthermore, pyroglutamic acid is a cyclized derivative of glutamic acid, which is formed non-enzymatically from glutamate and glutamine [53]. It is presumed that these metabolites are related to glutamine and glutamate metabolic pathways that are dramatically affected by Aβ_25–35_ injection and donepezil treatment. Similarly, taurine and glutamate levels in this study were the lowest in the Aβ + donepezil group. Taurine is present at high concentrations in the mammalian brain and plays several important roles in neurotransmission, neuromodulation, osmotic regulation, calcium uptake regulation, and cellular excitability [54]. Taurine can cross the blood–brain barrier and protect against glutamate toxicity. Increased dietary taurine has been hypothesized to improve cognitive function; however, the mechanisms of such protection have not been fully elucidated. Santa-Maria et al. [55] reported that taurine is a weak inhibitor of amyloid peptide aggregation. Based on these results, we suggest that glutamine and glutamate metabolism, taurine and hypotaurine metabolism, and the metabolites (glutamate, pyroglutamate, and taurine) belonging to these metabolic pathways may be potential markers for the early diagnosis of AD.

This study has several limitations. In this study, we created an Aβ-induced cognitive impairment model by injecting Aβ into the mouse’s ICV. However, since AD is not caused by a single factor but rather intertwined with factors such as the presence of Aβ oligomers, diet, aging, tau hyperphosphorylation, oxidative stress, and excitotoxicity, an Aβ-induced cognitive impairment mouse model cannot reproduce complete AD. Therefore, although the results of this study cannot be applied to the pathology of AD itself, it can provide basic data on the effects of Aβ or donepezil on gut microbiota and metabolites. Another limitation of the current study is the absence of a group of mice treated only with donepezil. In order to determine whether the effect of donepezil on the gut microbiota is effective only in the Aβ-induced cognitive impairment mouse model, it is necessary to compare it with the normal group treated only with donepezil. Therefore, further studies are needed to compare the effects of only the donepezil-treated group and the Aβ + donepezil-treated group to examine the effects of donepezil on gut microbiota and metabolites. Finally, in some cases, changes in gut microbiota or metabolites were not reversed by donepezil administration. This may indicate that it is not related to its therapeutic effect.

AD has a long onset process, with no specific method for early diagnosis. However, recent studies have shown that AD and the brain–gut axis are closely related. Our findings suggest that alterations in the gut microbiota might influence the induction and amelioration of Aβ-induced cognitive dysfunction via the gut–brain axis. In this study, the Aβ + donepezil group showed a significantly higher relative abundance of Verrucomicrobia than the Aβ group. The relative abundance of 12 taxa, including *Blautia* and *Akkermansia*, differed significantly between the groups. The major metabolic pathways in the brain tissues and gut microbiota affected by Aβ or donepezil treatment were related to amino acid pathways and sugar metabolism, respectively. This study could provide basic data on the effects of Aβ and donepezil on gut microbiota and metabolites in an Aβ-induced cognitive impairment mouse model.

## 4. Materials and Methods

### 4.1. Animals

Institute for Cancer Research mice (Male; age: 5–6 weeks; body weight: 25–30 g; Swiss CD-1 mice) were obtained from DehanBiolink Co. (Eumseong, Korea) and housed two mice per cage with specific pathogen-free conditions (temperature: 22–26 °C; relative humidity: 50–60%) with a 12-h light/dark cycle with free access to standard mouse food (Sangyang Co., Osen, Korea) and water. The mice were acclimatized for five days before the experiment. All behavioral experiments were conducted under the same ambient conditions in accordance with the Guide for Care and Use of Laboratory Animals of the National Research Council (NRC, 1996) and were approved by the Committee of Animal Care and Experiment of Dongshin University, Korea (DSU2019-04-02).

### 4.2. In Vivo Administration

Aβ_25–35_ aggregation and ICV injections were performed as previously described [56]. A total of 5 μL of Aβ_25–35_ solution (PBS containing aggregated Aβ_25–35_, 1 μg/μL, Sigma–Aldrich, St. Louis, MO, USA) or phosphate-buffered saline (PBS) was injected into the right ventricle using a 28-gauge needle with the following stereotaxic coordinates (mm) from bregma A: –0.22, L: 1.0, V: 2.5. Initially, to confirm the accurate ICV target of the intended ventricle position, tryptophan blue ICV injections were administered, and calibrations were made before the actual experiment. Mice were categorized into the following groups according to treatment: (1) control group (*n* = 6): PBS ICV + PBS PO; (2) Aβ-treated group (*n* = 6): Aβ_25–35_ ICV + PBS PO (Aβ); (3) donepezil-treated group (*n* = 6): Aβ_25–35_ ICV + donepezil (3 mg/kg PO) (Aβ + donepezil) [57]. On day 1, control group animals were injected with 5 μL PBS solution, whereas two others were injected with 5 µL containing 5 µg of Aβ_25–35_ (PBS containing aggregated 1 μg/μL of Aβ_25–35_). AD-induced mice were orally administrated (PO) with 3 mg/kg donepezil hydrochloride on days 3, 5, 7, and 9.

### 4.3. MWM Test

The MWM test was used to evaluate the effects of donepezil on mice's spatial learning and memory, as previously described, with minor modifications [58]. MWM equipment consisted of a circular black water tank (diameter: 120 cm; height: 50 cm) surrounded by various visual cues (stars, squares, rectangles, and circles) on pillars in fixed positions during the entire experiment. The tank was filled with tap water at a temperature of 22 ± 2 °C. The tank was virtually separated into four equal quadrants: southeast, northeast, southwest, and northwest. A white platform (diameter: 10 cm; height: 25 cm) was centered in the northwest quadrant. The experimental procedure included adaptive training (day 6, three times a day), hidden platform tests (days 7–10, two trials per day), and a spatial probe trial (immediately after the last hidden platform test on day 10, once daily).

### 4.4. Collection of Animal Brain Tissue and Feces

After the MWM experiment, on day 11, all mice were anesthetized, their chests were opened, and euthanasia was performed via cardiac puncture. For metabolite analysis, the hippocampus was immediately collected from the brain. The hippocampi of animals that were used for immunofluorescence analysis were perfused with ice-cold 4% paraformaldehyde before whole brain collection. On day 11, individual mice were placed in clean cages for 10–15 min to collect fecal samples. Each fecal pellet of mice was then placed in a sterile 1.7 mL Eppendorf tube and immediately frozen at −80°C.

### 4.5. Doublecortin Immunofluorescence Staining

The brains were post-fixed overnight before being incubated twice in 30% sucrose in PBS for 24 h at 2 °C. The brains were then frozen and cut into 30 μm sagittal sections using a cryostat. The slides were blocked with 6% bovine calf serum, incubated with doublecortin primary antibody (ratio 1:200 in cell staining buffer, 2 h) and then with Alexa Fluor 488 secondary antibody (ratio 490/525 nm, 1:200 in cell staining buffer, 2 h). The hippocampal dentate gyrus area was captured using an Invitrogen EVOS FL Auto Imaging System using the GFP channel –Ex/Em = 490/525 nm (Thermo Fisher Scientific, Waltham, MA, USA). Doublecortin-positive neurons were counted in a 100 µm-wide area across all tissue sections.

### 4.6. Cell Culturing and Donepezil Treatment

HT-22 mouse hippocampal cells were chosen for the study. The culturing media conditions are: Dulbecco’s modified Eagle’s medium supplemented with fetal bovine serum (10%) and penicillin–streptomycin (1%); incubator parameters: 5% CO_2_ and 37 °C. Cells were cultured at a density of 1 × 10^4^ cells per well (with 0.1 mL media) in 96-well plates to test the cell availability of donepezil. The amount of donepezil (3 µM) was determined according to the screening for the maximum safe dose of donepezil and the protective effect of donepezil against Aβ_25–35_ stress (7 µM) induced in HT-22 cells (Figure S3). HT-22 cell treatment was grouped as an Aβ + low donepezil (0.3 µM) group and an Aβ + high donepezil (3 µM) group.

### 4.7. DNA Extraction and 16S rRNA Gene Amplicon Sequencing

Fecal samples were collected from all mice upon defecation and stored at –80 °C for subsequent analysis. For DNA extraction, 100 mg of fecal sample was extracted using the AccuFAST automation system (AccuGene, Incheon, Korea) in accordance with the manufacturer’s instructions. For MiSeq sequencing, bacterial genomic DNA amplification was performed using primers of 515 and 806 bp containing Nextera adaptor sequences, targeting the V4 hypervariable region of the 16S rRNA genes [59]. With KAPA HiFi HotStart ReadyMix, 16S rRNA genes were amplified in 25 polymerase chain reactions (PCRs) (Roche, Pleasanton, CA, USA). The resulting PCR products (approximately 250 bp) were purified using HiAccuBeads (AccuGene). Using the MiS2eq Reagent Kit v2 for 500 cycles (Illumina, San Diego, CA, USA), amplicon libraries at an equimolar ratio were pooled. Pooled libraries were sequenced using an Illumina MiSeq system. For the raw data sets, raw sequencing reads were subjected to reference-based chimeric filtering using VSEARCH v2.10.3 [60]. The chimeric filtered sequences were assigned to operational taxonomic units (OTUs) using OTU picking in the QIIME pipeline. The sequences were clustered using UCLUST into OTUs based on the SILVA 132 database (arb-silva.de/documentation/release-132) (pre-clustered at 97% similarity threshold).

### 4.8. Metabolites Extraction from Cells, Brain Tissue, and Feces

The cultured cells were washed with 0.9% NaCl (2 °C). Next, 300 µL of methanol (−20 °C) was added to the well surface and incubated for 1 min, after which 300 µL of distilled water (2 °C) was added to the wells on ice. Each well of the plate was scraped off using a cell scraper. The cell–liquid mixture was mixed by pipetting up and down and then transferred (600 µL) to an Eppendorf tube containing 300 µL of cold chloroform (−20 °C). The mixture was vortexed for 5 min at 4 °C and centrifuged at 13,000 rpm for 5 min at 2 °C. Without touching the interphase, 100 μL of the polar (upper) phase was transferred to a vial and sent for metabolite analysis. After the brain tissues were homogenized, 4 mL of methanol and 4 mL of chloroform were added to 350 mg of the brain tissue and extracted on ice for 15 min. After centrifuging the sample at 13,000 rpm for 5 min at 4 °C, 100 µL of supernatant was pooled for metabolite analysis. For fecal metabolite analysis, 70 mg of fecal samples were extracted after lyophilization by mixing with 500 μL of ice-cold 95% methanol. The mixture was vortexed for 2 min, sonicated, and centrifuged (13,000 rpm, 4 °C, 15 min).

### 4.9. Sample Derivatization and Metabolite Analysis

A total of 200 microliters of cold methanol was added to 100 μL of each supernatant to precipitate the protein. The mixture was vortexed and centrifuged, and 100 µL of the supernatant was freeze-dried. For fecal samples, 300 μL of the supernatant of the fecal extract was freeze-dried. After freeze-drying, 80 µL of O-methoxyamine hydrochloride (20 mg/mL) in pyridine solution was added to each freeze-dried sample. The samples were vortex-mixed for 30 s and incubated at 30 °C for 90 min in the dark. Thirty µL of N-methyl-N-trimethylsilyl-trifluoroacetamide with 1% trimethylchlorosilane was added to each sample for silylation, followed by vortexing for 30 s and incubation at 37 °C for 30 min. Approximately 10 µL of ribitol (0.5 mg/L) was used as the internal standard. The derivatized samples were analyzed using a gas chromatography-mass spectrometry (GC-MS) system (QP2020; Shimadzu, Kyoto, Japan). An Rtx-5MS fused silica capillary column (30 m × 0.25 mm, 0.25 µm; J&W Scientific, Folsom, CA, USA) was used for the separation of metabolites. The front-inlet temperature was set to 230 °C. The column temperature was maintained at 80 °C for 2 min isothermally, raised by 15 °C/min to 330 °C, and held there for 6 min isothermally. The transfer line and ion source temperatures were 250 and 200 °C, respectively. Ionization was achieved using a 70 eV electron beam. The flow rate of helium gas through the column was 1 mL/min. Approximately 20 scans/s were recorded in the mass range 85–500 m/z. A GC solution (Shimadzu, Kyoto, Japan) was used to obtain the chromatograms and mass spectra. The stability and performance of the instrument were measured, along with the reproducibility of the sample treatment procedure. Quality control was assessed for every ten samples during the run.

### 4.10. Data Processing and Multivariate Analysis

The GC-MS data were converted to a netCDF file and processed using the MetAlign software (wur.nl/nl/show/metalign.htm) for peak detection and alignment [61]. MetAlign parameters were set according to the AIoutput scaling requirements: a peak slope factor of 2, peak threshold of 10, average peak width at half height of 25, and peak threshold factor of 4. These settings corresponded to a retention time of 3–26 min and a mass range of 85–500 m/z. The data were imported into AIoutput software for peak prediction and identification [62]. After normalization using an internal standard (retention time 11.205 min, m/z 147), multivariate statistical analyses were performed. To visualize the variance of metabolites, principal component analysis (PCA) and partial least squares discriminant analysis (PLS-DA) of the GC-MS data were performed using SIMCA-P (version 15.0; Umetrics, Umea, Sweden). For model validation, a 200-fold cross-validation was performed. Metabolites were identified by comparing their mass spectra with those of AIoutput software, the NIST library, and the human metabolome database.

### 4.11. Statistical Analysis

Statistical analyses were performed using GraphPad Prism 9.3.1 (Graphpad, San Diego, CA, USA). All data are expressed as the mean ± standard deviation. Statistical significance was set at *p* < 0.05. One-way analysis of variance (ANOVA) followed by Tukey’s posthoc test was used to evaluate the statistical significance of differences among three or more groups. The associations among the metabolites in the brain samples, feces, and microorganisms in feces were assessed using Spearman’s rank correlation analysis. An FDR-corrected *p*-value < 0.05 was calculated using the Benjamini and Hochberg method.

### 4.12. Metabolic Pathway Analysis

Metabolic pathway analysis was performed using MetaboAnalyst web software (metaboanalyst.ca access date: 31 August 2022) and Kyoto Encyclopedia of Genes and Genomes by filtering the dataset using the FDR-adjusted *p*-value < 0.05 and impact value > 0.1 to reveal how significant metabolites are involved in different pathways [63].

## Data Availability

The data presented in this study are available on request from the corresponding author.

## Supplementary Materials

The following supporting information can be downloaded at: https://www.mdpi.com/article/10.3390/molecules27196591/s1, Figure S1: PLS-DA score plot derived from the GC-MS data.; Figure S2: Metabolic pathways in brain tissues and in feces affected by each treatment.; Figure S3: Screening for donepezil’s maximum safe dosage and protective effect against Aβ_25–35_ stress induced in HT22 cells. Table S1: Relative abundance profile of bacterial communities.
