# Supplementary material for: Effects of Donepezil Treatment on Brain Metabolites, Gut Microbiota, and Gut Metabolites in an Amyloid Beta-Induced Cognitive Impairment Mouse Pilot Model
Source: Molecules. 2022 Oct 5;27(19):6591. doi: 10.3390/molecules27196591 (PMC9572896; doi:10.3390/molecules27196591)
Supplement: Supplementary file 1 [file molecules-27-06591-s001.zip › molecules-1918382-supplementary.pdf]

Supplementary Material

Table S1. Relative abundance profile of bacterial communities at the phylum, genus, and species level

	Control (n = 6)	Aβ (n = 6)	Aβ+Donepezil (n = 6)
Relative abundance at phylum level (%, mean ±SD)			
Actinobacteria	0.03±0.01	0.05±0.07	0.01±0.01
Bacteroidetes	70.08±16.20	63.72±21.03	71.70±11.51
Cyanobacteria	0.57±0.49	0.36±0.25**	1.42±0.77**
Deferribacteres	0.61±0.53	0.79±1.11	0.46±0.67
Firmicutes	27.66±15.06	33.17±19.50	20.93±8.92
Patescibacteria	0.06±.09	0.07±0.08	0.03±0.03
Proteobacteria	0.45±0.16	0.62±0.43	0.80±0.83
Tenericutes	0.49±0.20	0.99±0.51	0.56±0.35
Verrucomicrobia	0.01±0.02	0.14±0.14**	3.96±3.21**
Bacteria_Other	0.04±0.03	0.07±0.04	0.12±0.07
Unclassified_Other	0.00±0.00	0.01±0.01	0.01±0.01
Relative abundance at genus level (%, mean ±SD)			
Bacteroides	2.53±1.42	3.44±2.68	6.18±2.48*
Odoribacter	1.14±0.33	1.40±0.41	1.15±0.33
Muribaculaceae__uncultured Bacteroidales bacterium	15.39±2.93	9.84±4.21*	9.21±2.36*
Muribaculaceae__uncultured bacterium	32.91±13.93	30.65±13.58	34.73±7.76
Muribaculaceae__uncultured organism	0.97±1.04	0.53±0.60	0.63±0.33
Muribaculaceae_Other	1.23±0.87	3.24±3.16	2.04±1.53
Prevotellaceae UCG-001	2.31±1.69	4.74±1.76	8.17±2.48***#
Alistipes	12.49±7.69	8.89±2.73	8.61±3.17
Rikenellaceae RC9 gut group	0.83±0.40	0.55±0.70	0.38±0.20
Parabacteroides	0.27±0.08	0.45±0.16	0.58±0.23*
Gastranaerophilales__uncultured bacterium	0.26±0.13	0.14±0.09	0.54±0.25*##
Gastranaerophilales__uncultured rumen bacterium	0.31±0.39	0.22±0.16	0.69±0.33#
Mucispirillum	0.61±0.53	0.79±1.11	0.46±0.67
Lactobacillus	0.02±0.02	0.14±0.16	0.07±0.07
Clostridiales bacterium enrichment culture clone 06-1235251-67	0.15±0.12	0.03±0.01	0.15±0.20
Clostridiales vadinBB60 group__uncultured bacterium	1.67±1.33	0.54±0.19	0.97±0.69
Lachnospiraceae__ASF356	0.05±0.04	0.15±0.21	0.06±0.06
Blautia	0.10±.0.06	0.04±0.02*	0.01±0.01**
Lachnospiraceae_GCA-900066575	0.04±0.03	0.13±0.12	0.04±0.03
Lachnoclostridium	0.25±0.11	0.60±0.40	0.22±0.11#
Lachnospiraceae NK4A136 group	9.67±5.34	10.72±7.79	5.34±3.01
Lachnospiraceae UCG-001	0.94±1.49	0.40±0.28	0.09±0.05
Roseburia	0.55±0.60	1.05±0.57	0.33±0.27
Tyzzerella	0.10±0.09	0.11±0.11	0.05±0.05
Tyzzerella 3	0.10±0.08	0.08±0.06	0.03±0.02
[Eubacterium] ventriosum group	0.24±0.07	0.11±0.04	0.22±0.21
[Eubacterium] xylanophilum group	0.08±0.05	0.24±0.20	0.08±0.07
Lachnospiraceae__uncultured	1.18±1.29	1.18±0.72	0.39±0.33
Lachnospiraceae_Other	2.74±1.71	6.44±5.56	3.26±2.68
Peptococcaceae__uncultured	0.43±0.28	0.62±0.33	0.86±0.36

Anaerotruncus	0.32±0.20	0.40±0.24	0.25±0.18
Butyricicoccus	0.23±0.23	0.70±0.86	0.07±0.05
Intestinimonas	0.16±0.09	0.43±0.38	0.20±0.15
Oscillibacter	1.20±1.03	1.31±1.23	0.76±0.78
Oscillospira	0.18±0.19	0.22±0.21	0.10±0.11
Ruminiclostridium	1.24±1.50	1.06±0.91	0.60±0.51
Ruminiclostridium 5	0.09±0.06	0.13±0.10	0.04±0.03
Ruminiclostridium 9	0.92±0.61	1.09±0.67	0.54±0.52
Ruminococcaceae NK4A214 group	0.07±0.03	0.09±0.03	0.11±0.05
Ruminococcaceae UCG-014	1.95±1.05	2.39±1.11	4.04±1.29*
Ruminococcus 1	0.97±0.59	0.50±0.19	0.35±0.21*
Ruminococcaceae__UBA1819	0.05±0.05	0.13±0.16	0.02±0.03
Ruminococcaceae__uncultured	0.87±0.79	0.87±0.86	0.60±0.89
Ruminococcaceae_Other	0.32±0.26	0.47±0.42	0.20±0.20
Erysipelotrichaceae__uncultured	0.08±0.04	0.10±0.05	0.09±0.03
Desulfovibrionaceae__uncultured	0.36±0.18	0.51±0.46	0.65±0.88
Parasutterella	0.06±0.04	0.10±0.17	0.06±0.11
Anaeroplasma	0.12±0.11	0.24±0.53	0.09±0.09
Mollicutes RF39__uncultured bacterium	0.15±0.17	0.16±0.10	0.13±0.06
Mollicutes RF39__unidentified	0.03±0.01	0.05±0.02	0.22±0.27
Mollicutes RF39;Other	0.14±0.03	0.44±0.09****	0.06±0.02$^{\#\#\#\#}$
Akkermansia	0.01±0.02	0.14±0.14**	3.96±3.21$^{\#\#}$
Bacteria__Other	0.04±0.03	0.07±0.04	0.12±0.07
Cut off (<0.1%)	0.84±0.83	0.93±0.95	1.21±1.59
Relative abundance at species level (%, mean ±SD)			
Bacteroides ovatus V975	0.45±0.14	0.48±0.13	0.69±0.24
Bacteroides__unidentified	0.93±0.76	1.29±0.60	2.60±2.09
Bacteroides__Other	0.77±0.42	1.65±2.19	2.88±1.47
Odoribacter__uncultured bacterium	1.07±0.32	1.40±0.41	1.15±0.33
Muribaculaceae__uncultured Bacteroidales bacterium	15.29±3.26	9.84±4.21	9.24±2.36
Muribaculaceae__uncultured bacterium	34.55±14.90	30.65±13.58	34.73±7.76
Muribaculaceae__uncultured organism	1.14±1.07	0.53±0.60	0.63±.33
Muribaculaceae__Other	1.43±0.81	3.24±3.16	2.04±1.53
Prevotellaceae__uncultured Bacteroidales bacterium	2.60±1.72	4.74±1.76	8.17±2.48
Alistipes__uncultured bacterium	10.53±6.93	8.83±2.79	8.44±3.23
Alistipes__Other	0.05±0.04	0.06±0.09	0.15±0.09
Rikenellaceae RC9 gut group__uncultured bacterium	0.74±0.37	0.55±0.70	0.38±0.20
Parabacteroides goldsteinii CL02T12C30	0.21±0.10	0.43±0.17	0.39±0.12
Parabacteroides__Other	0.07±0.03	0.02±0.02	0.19±0.19
Candidatus Gastranaerophilales bacterium Zag_111	0.00±0.00	0.00±0.00	0.20±0.34
Gastranaerophilales__uncultured bacterium	0.24±0.14	0.14±0.09	0.54±0.25
Gastranaerophilales__uncultured rumen bacterium	0.32±0.43	0.22±0.16	0.69±0.33$^{\#}$
Mucispirillum sp. 69	0.49±0.48	0.79±1.11	0.46±0.67
Lactobacillus murinus	0.03±0.02	0.14±0.16	0.54±0.25
Clostridiales bacterium enrichment culture clone 06-1235251-67	0.10±.0.06	0.03±.01	0.15±0.20
Clostridiales vadinBB60 group__uncultured bacterium	1.19±0.65	0.54±0.19	0.97±0.69
Clostridium sp. ASF356	0.04±0.04	0.15±0.21	0.05±0.05
Coprococcus 2__uncultured organism	0.00±0.00	0.00±0.00	0.16±0.28
Lachnospiraceae__GCA-900066575 uncultured bacterium	0.04±0.03	0.13±0.12	0.04±0.03
Lachnoclostridium__uncultured bacterium	0.17±0.08	0.36±0.24	0.09±0.05
Lachnoclostridium__uncultured organism	0.05±0.06	0.24±0.20	0.13±0.11
Lachnospiraceae bacterium COE1	0.62±0.40	0.22±0.19	0.38±0.22
Lachnospiraceae NK4A136 group__uncultured Clostridiales bacterium	5.91±3.26	6.60±5.11	3.70±2.27
Lachnospiraceae NK4A136 group__uncultured bacterium	3.01±2.47	3.63±2.62	1.08±0.89

Lachnospiraceae NK4A136 group__unidentified	3.01±2.47	3.63±2.62	1.08±0.89
Lachnospiraceae UCG-001__uncultured bacterium	1.03±1.59	0.32±0.23	0.04±0.03
Roseburia__uncultured bacterium	0.55±0.61	0.97±0.55	0.31±0.27
Tyzzerella__uncultured bacterium	0.10±0.10	0.11±0.11	0.05±0.05
[Eubacterium] ventriosum group__uncultured bacterium	0.23±0.07	0.11±0.04	0.22±0.21
[Eubacterium] xylanophilum group__uncultured bacterium	0.09±0.06	0.24±0.20	0.08±0.07
Lachnospiraceae__uncultured bacterium	0.24±0.24	0.25±0.19	0.08±0.07
Lachnospiraceae__uncultured organism	0.22±0.36	0.39±0.42	0.06±0.07
Lachnospiraceae__uncultured	0.71±0.76	0.41±0.30	0.20±0.21
Lachnospiraceae__Other	2.79±1.91	6.44±5.56	3.26±2.68
Peptococcaceae__uncultured bacterium	0.31±0.33	0.51±0.35	0.77±0.36
Peptococcaceae__uncultured	0.13±0.07	0.11±0.04	0.09±0.06
Anaerotruncus__uncultured bacterium	0.32±0.22	0.37±0.23	0.25±0.18
Butyricicoccus__uncultured bacterium	0.25±0.25	0.70±0.86	0.07±0.05
Intestinimonas__uncultured bacterium	0.17±0.09	0.43±0.38	0.20±0.15
Oscillibacter__uncultured bacterium	0.55±0.60	0.39±0.45	0.33±0.39
Oscillibacter__unidentified	0.68±0.53	0.88±0.90	0.39±0.34
Oscillospira__uncultured bacterium	0.20±0.20	0.22±0.21	0.10±0.11
Ruminiclostridium__uncultured bacterium	1.36±1.62	0.99±0.82	0.51±0.43
Ruminiclostridium 9__uncultured bacterium	0.97±0.66	1.07±0.67	0.53±0.51
Ruminococcaceae UCG-014__uncultured Firmicutes bacterium	0.05±0.04	0.15±0.11	0.28±0.15
Ruminococcaceae UCG-014__uncultured bacterium	0.78±0.43	1.00±.27	2.21±1.20
Ruminococcaceae UCG-014__uncultured rumen bacterium	0.31±0.11	0.66±0.39	0.77±0.51
Ruminococcaceae UCG-014__Other	0.74±0.80	0.53±0.34	0.72±0.30
Ruminococcus sp. UNK.MGS-30	0.43±0.68	0.01±0.01	0.07±0.09
Ruminococcus 1__metagenome	0.33±0.30	0.13±0.09	0.02±0.05
Ruminococcus 1__uncultured bacterium	0.34±0.27	0.37±0.21	0.26±0.20
Ruminococcaceae__UBA1819 uncultured bacterium	0.04±0.05	0.13±0.16	0.02±0.03
Clostridium sp. Culture-1	0.82±0.83	0.76±0.78	0.52±0.82
Ruminococcaceae__unidentified	0.10±0.06	0.11±0.08	0.07±0.06
Ruminococcaceae__Other	0.35±0.28	0.47±0.42	0.20±0.20
Desulfovibrionaceae__uncultured bacterium	0.33±0.16	0.44±0.41	0.60±0.80
Anaeroplasma__uncultured bacterium	0.12±0.12	0.24±0.53	0.09±0.09
Mollicutes RF39__uncultured bacterium	0.18±0.18	0.16±0.10	0.13±0.06
Mollicutes RF39__Other	0.14±0.03	0.44±0.09****	0.06±0.02####
Akkermansia__uncultured bacterium	0.01±0.02	0.14±0.14	3.96±3.21**##
Bacteria__Other	0.04±0.03	0.07±0.04	0.12±0.07
Cut off (<0.1%)	1.90±1.74	2.29±2.43	1.98±2.42

Data are presented as mean±SD. **P*<0.05, ***P*<0.01, ****P*<0.001, *****P*<0.0001 vs. control groups; # *P*<0.05, ##*P*<0.01, ####*P*<0.0001 vs. Aβ group.

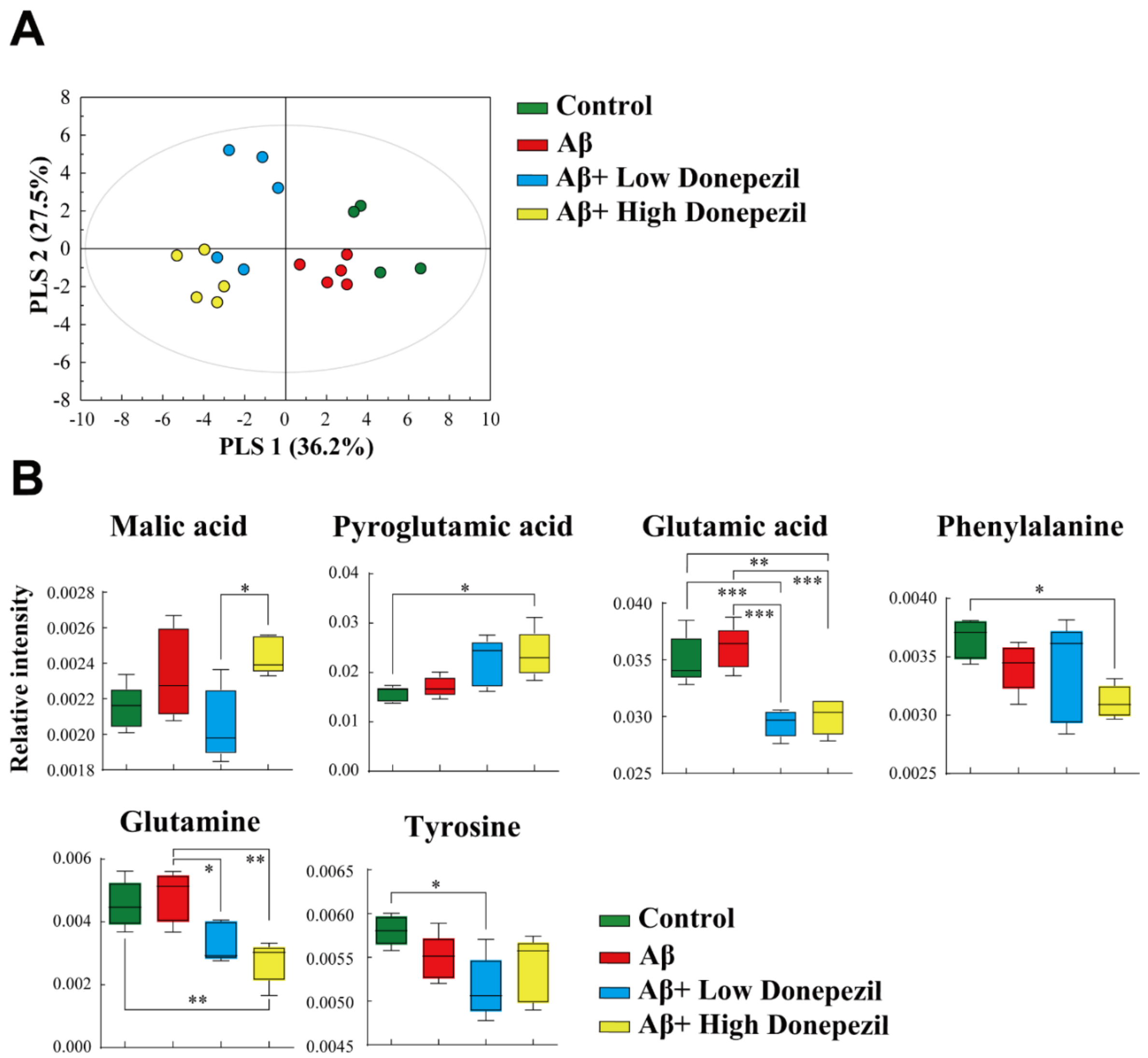

Supplementary Figure S1. (A) Supervised partial least squares discriminant analysis (PLS-DA) score plot derived from the gas chromatography-mass spectrometry (GC-MS) data of HT22 cells of the control, Aβ, Aβ + low donepezil (0.3 μM), and Aβ + high donepezil (3 μM) groups. (B) Box plots of significantly different metabolites in the feces of the control, Aβ, and Aβ + donepezil groups. *, $p < 0.05$; **, $p < 0.01$; ***, $p < 0.001$. A false discovery rate (FDR) of 5% was applied to all tests to correct for multiple testing.

A

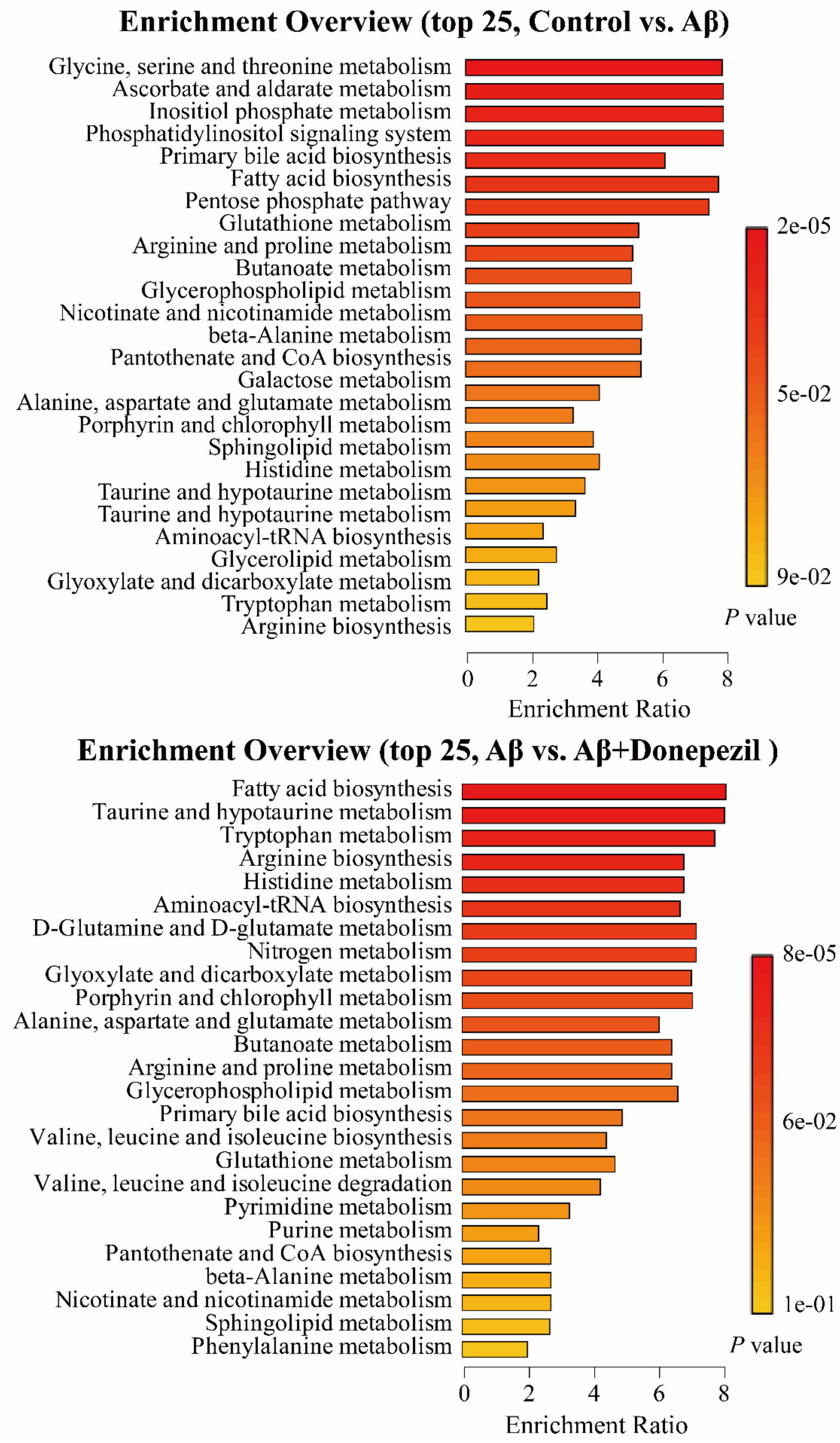
Enrichment Overview (top 25, Control vs. Aβ)
Glycine, serine and threonine metabolism
Ascorbate and aldarate metabolism
Inositiol phosphate metabolism
Phosphatidylinositol signaling system
Primary bile acid biosynthesis
Fatty acid biosynthesis
Pentose phosphate pathway
Glutathione metabolism
Arginine and proline metabolism
Butanoate metabolism
Glycerophospholipid metablism
Nicotinate and nicotinamide metabolism
beta-Alanine metabolism
Pantothenate and CoA biosynthesis
Galactose metabolism
Alanine, aspartate and glutamate metabolism
Porphyrin and chlorophyll metabolism
Sphingolipid metabolism
Histidine metabolism
Taurine and hypotaurine metabolism
Taurine and hypotaurine metabolism
Aminoacyl-tRNA biosynthesis
Glycerolipid metabolism
Glyoxylate and dicarboxylate metabolism
Tryptophan metabolism
Arginine biosynthesis
0
2
4
6
8
Enrichment Ratio
2e-05
5e-02
9e-02
P value
Enrichment Overview (top 25, Aβ vs. Aβ+Donepezil)
Fatty acid biosynthesis
Taurine and hypotaurine metabolism
Tryptophan metabolism
Arginine biosynthesis
Histidine metabolism
Aminoacyl-tRNA biosynthesis
D-Glutamine and D-glutamate metabolism
Nitrogen metabolism
Glyoxylate and dicarboxylate metabolism
Porphyrin and chlorophyll metabolism
Alanine, aspartate and glutamate metabolism
Butanoate metabolism
Arginine and proline metabolism
Glycerophospholipid metabolism
Primary bile acid biosynthesis
Valine, leucine and isoleucine biosynthesis
Glutathione metabolism
Valine, leucine and isoleucine degradation
Pyrimidine metabolism
Purine metabolism
Pantothenate and CoA biosynthesis
beta-Alanine metabolism
Nicotinate and nicotinamide metabolism
Sphingolipid metabolism
Phenylalanine metabolism
0
2
4
6
8
Enrichment Ratio
8e-05
6e-02
1e-01
P value

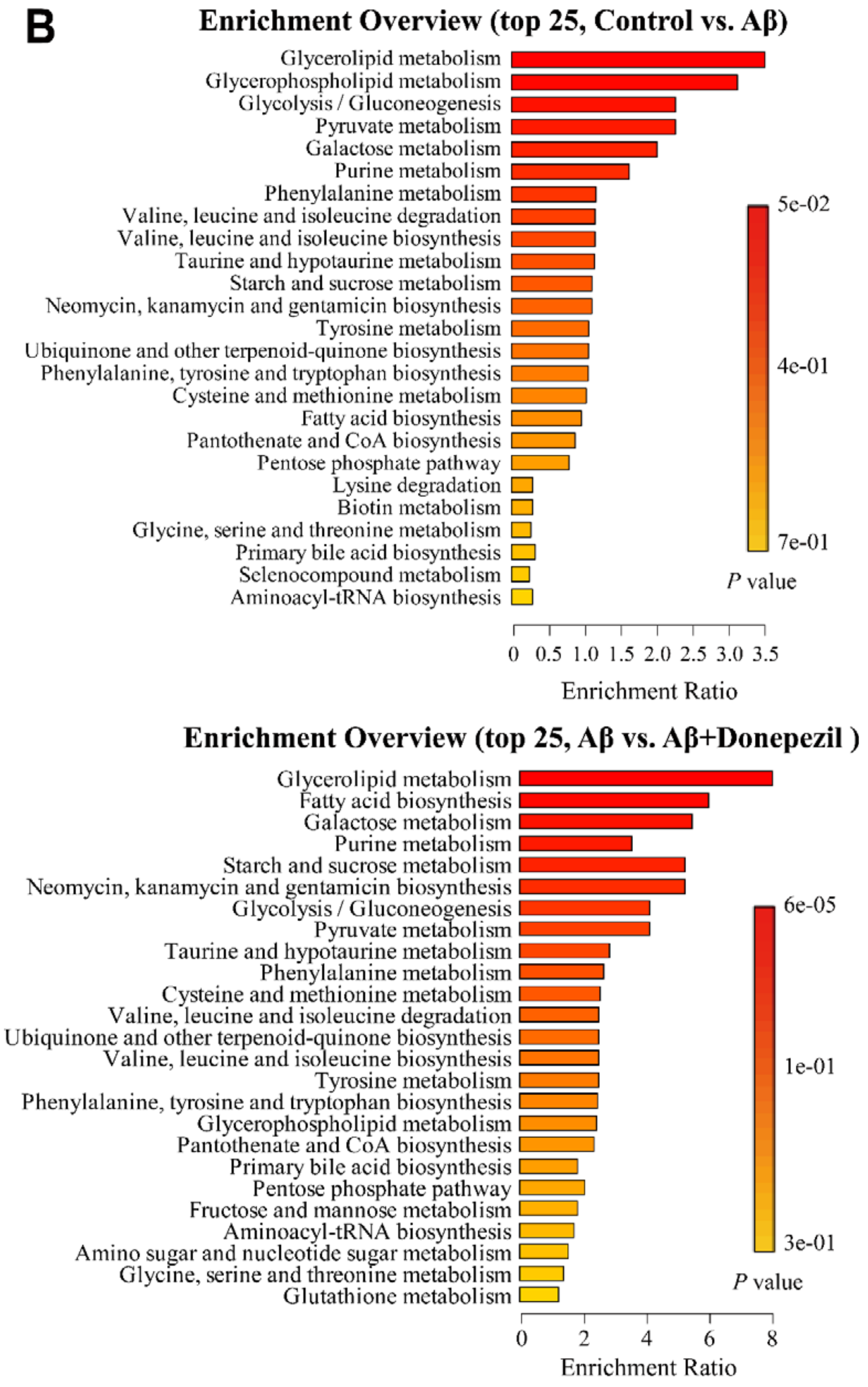

Supplementary Figure S2. (A) Metabolic pathways in brain tissues affected by each treatment. (B) Metabolic pathways in feces affected by each treatment.

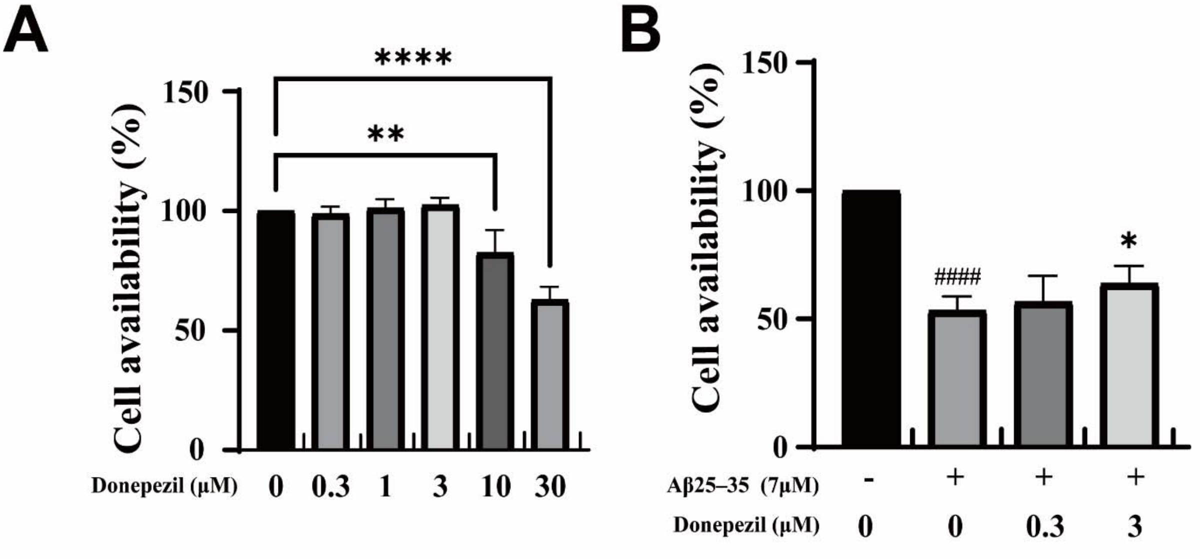

Supplementary Figure S3. Screening for donepezil maximum safety dosage and protective effect against amyloid β (Aβ)$_{25–35}$ stress induced in HT22 cells. (A) Screening for maximum donepezil safety concentration. The highest safety concentration of donepezil used in the experiment was determined to be 3 μM. To conduct this experiment, cells were seeded in a 96-well plate; after incubation for 24 h, donepezil at different concentrations was added, and cell availability was examined after 24 h of incubation. (B) Protective effect of donepezil against Aβ$_{25–35}$ stress. The result indicated that 3 μM of donepezil administration ameliorated the HT22 cell availability. To conduct this experiment, cells were seeded in a 96-well plate; after incubation for 24 h, donepezil from 0.3 and 3 μM was added 1 h before Aβ$_{25–35}$ (7 μM) challenge, and cell availability was examined after 24 h of incubation. Data are presented as the mean ± standard deviation values of triple determinations. #, $p < 0.05$; ##, $p < 0.01$; ###, $p < 0.001$; ####, $p < 0.0001$ vs. control group; *, $p < 0.05$; **, $p < 0.01$; ***, $p < 0.001$; ****, $p < 0.0001$ vs. amyloid β (Aβ) group.
